# The Kindlin2-p53-SerpinB2 signaling axis is required for cellular senescence in breast cancer

**DOI:** 10.1038/s41419-019-1774-z

**Published:** 2019-07-15

**Authors:** Khalid Sossey-Alaoui, Elzbieta Pluskota, Dorota Szpak, Edward F. Plow

**Affiliations:** 1 Department of Cardiovascular & Metabolic Sciences, Lerner Research Institute, Cleveland Clinic, Cleveland, OH USA; 2 Case Western Reserve University-MetroHealth Medical Research Center, Cleveland, OH USA

**Keywords:** Histocytochemistry, Immunochemistry, Mechanisms of disease, Breast cancer

## Abstract

In cancer, cellular senescence is a complex process that leads to inhibition of proliferation of cells that may develop a neoplastic phenotype. A plethora of signaling pathways, when dysregulated, have been shown to elicit a senescence response. Two well-known tumor suppressor pathways, controlled by the p53 and retinoblastoma proteins, have been implicated in maintaining the cellular senescence phenotype. Kindlin-2, a member of an actin cytoskeleton organizing and integrin activator proteins, has been shown to play a key role in the regulation of several hallmarks of several cancers, including breast cancer (BC). The molecular mechanisms whereby Kindlin-2 regulates cellular senescence in BC tumors remains largely unknown. Here we show that Kindlin-2 regulates cellular senescence in part through its interaction with p53, whereby it regulates the expression of the p53-responsive genes; i.e., SerpinB2 and p21, during the induction of senescence. Our data show that knockout of Kindlin-2 via CRISPR/Cas9 in several BC cell lines significantly increases expression levels of both SerpinB2 and p21 resulting in the activation of hallmarks of cellular senescence. Mechanistically, interaction between Kindlin-2 and p53 at the promotor level is critical for the regulated expression of SerpinB2 and p21. These findings identify a previously unknown Kindlin-2/p53/SerpinB2 signaling axis that regulates cellular senescence and intervention in this axis may serve as a new therapeutic window for BCs treatment.

## Introduction

Physiological cellular senescence is defined as the irreversible arrest of cell proliferation^1^. In the context of cancer, senescence leads to inhibition of cell proliferation and, thereby, can suppress progression of the neoplastic phenotype^2^. A plethora of signaling pathways, when dysregulated, have been identified to elicit a senescence response^3^. These include DNA damage, dysfunctional telomeres, perturbations to chromatin organization, activation of certain oncogenes, and inhibition of certain tumor suppressor genes^2–4^. In both physiological and pathological settings, senescence is regulated by at least two well-defined pathways; i.e., the p53/p21 and p16INK4a/pRB pathways^3^. Although these two pathways are activated by different stimuli, they interact and cooperate to control senescence^5^. This study implicates kindlin-2 in the p53/p21 senescence pathway.

Kindlin-2 (FERMT2) is one of three kindlin family members of FERM domain-containing proteins. It is an adapter protein with many different binding partners including the cytoplasmic tail of integrin beta subunits, an interaction that permits kindlins to function as key regulators in integrin activation^6–8^. Several recent studies have associated Kindlin-2 with the pathology of cancers originating from different organs, including gastric, prostate and breast cancer (BC)^9,10^. In BC, Kindlin-2 is involved in the regulation of the tumor microenvironment by influencing the recruitment of macrophages to the tumor and their polarization into a pro-tumorigenic phenotype^11–13^. We have also established Kindlin-2 as a major regulator of the EMT process in BC^14^ and associated Kindlin-2 with pathways involved in chemo- and radio-resistance of BC cells^15^. However, the role of Kindlin-2 in senescence has not received any attention.

In the present study, we define a new role for Kindlin-2 in the regulation of senescence in BC. RNA-Seq analyses determined that loss of Kindlin-2 expression in BC cell lines resulted in a significant increase in SerpinB2 and p21 expression, both known to be associated with cancer cell senescence^16,17^. Concomitant with the increase of SerpinB2 and p21 expression, a significant increase of β-galactosidase staining, a marker for senescence, was observed. Further investigations revealed that binding of Kindlin-2 to p53, a senescence-inducer gene, inhibits the binding of p53 to the promoter of SerpinB2 and p21. Importantly, in the Kindlin-2-deficient cancer cells, the binding of p53 to the promoters of SerpinB2 and p21 was significantly enhanced, thereby identifying a novel role of Kindlin-2 in the regulation of BC progression and metastasis.

## Results

### *Loss of Kindlin-2 induces BC senescence by activating SerpinB2 in vitro and in vivo*

**Fig. 1:**
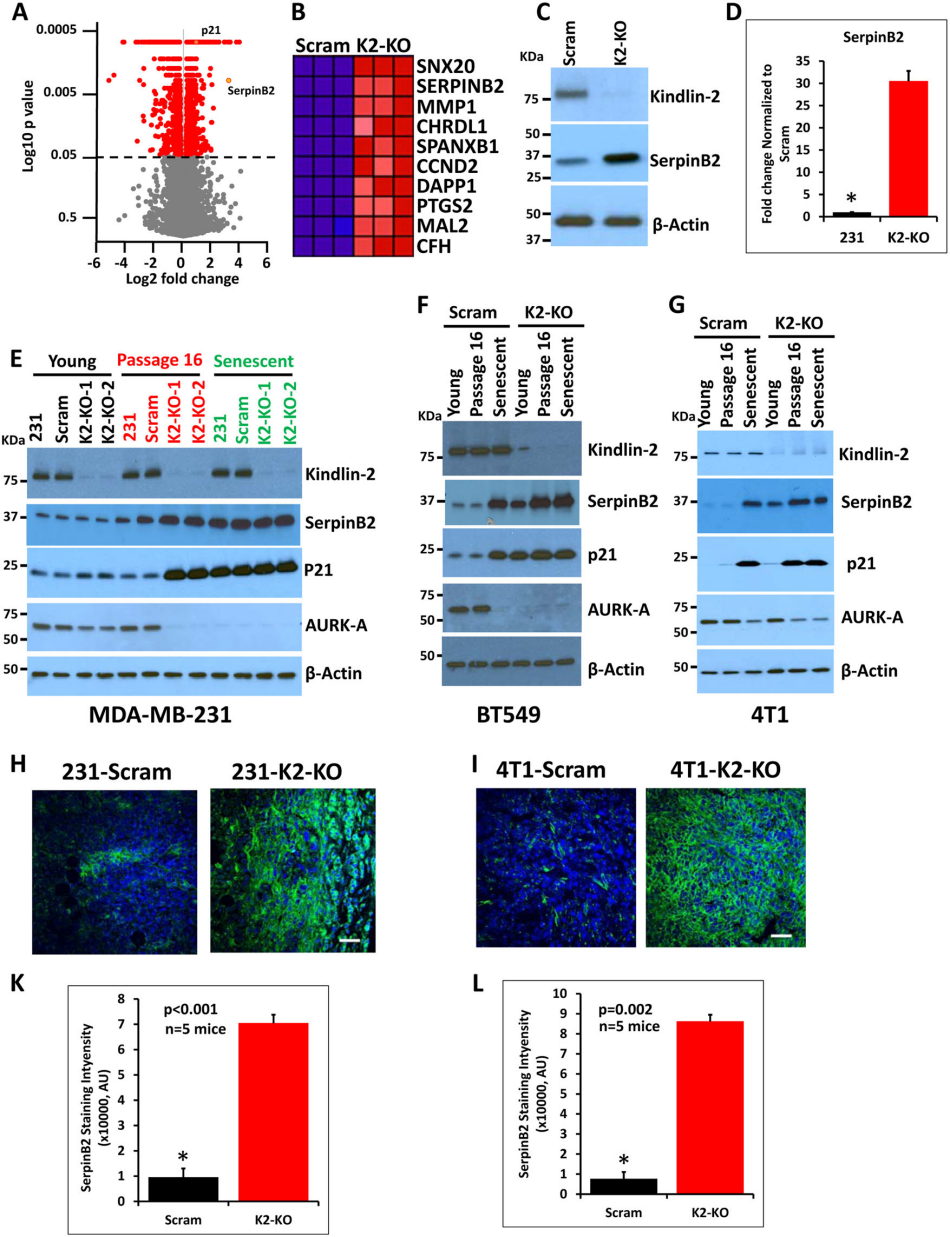
Loss of Kindlin-2 induces breast cancer cell senescence by activating SerpinB2 *in vitro* and *in vivo*. **a** Volcano plot from the RNA-seq analysis of the differentially expressed genes between control (Scram) and pooled cell populations of Kindlin-2-deficient (KO-KO) MDA-MB-231 cells. The X-axis represents the Log2 fold change in expression levels and the Y-axis shows the p values. p21 and SerpinB2 are shown in orange dots. **b** Heat-map showing the top 10 differentially expressed genes between control (Scram) and Kindlin-2-deficient (K2-KO) MDA-MB-231 cells (Passage 16). **c** Western blots with anti-kindlin-2 (top panel) and anti-SerpinB2 (middle panel) on lysates of control (Scram) and Kindlin-2-deficient (K2-KO) MDA-MB-231 cells. Anti-β-Actin (lower panel) was used as a loading control. **d** Quantitative real-time RT-PCR of SerpinB2 transcripts in control (Scram) and Kindlin-2-deficient (K2-KO) MDA-MB-231 cells. Data are representative of 3 independent experiments (ns, not significant); *, **p < 0.05, student *t*-test. **e-g** Western blots with the indicated antibodies on lysates of parental (231), control (Scram) and Kindlin-2-deficient (K2-KO) MDA-MB-231 **e**, BT549 **f** or 4T1 cells **g** at passage 3 or less (Young), passage 16 or treated with 1 mM JQ1 for 10 days (Senescent). Anti-β-Actin was used as a loading control. **h**, **i** Representative images of control (Scram) or Kindlin-2-deficient (K2-KO) MDA-MB-231 **h** or 4T1 **i** -derived tumor sections stained for SerpinB2 (green). Nuclei were counterstained with DAPI. Scale bar, 146 μm. **k**, **l** Quantification of SerpinB2 staining areas in MDA-MB-231 **k** and 4T1 **l**. Data are expressed as mean ± SEM. *p < 0.005; *n* = 5 mice

Several published studies implicated Kindlin-2 in the development, progression and metastasis of BC^11,14,15^. To further explore the role of Kindlin-2 in BC pathology, we performed an RNA-seq analysis to identify genes affected by loss of Kindlin-2. We used CRISPR to efficiently target and knockout (KO) Kindlin-2 in MDA-MB-231 BC cells^11,14^. RNA-seq analysis was performed on the Kindlin-2-deficient MDA-MB-231 cells (K2-KO) and controls with a scrambled sgRNA (Scram)^11,14^. A volcano plot (Fig. 1a) identified SerpinB2 as one of the top genes significantly upregulated (32-fold increase, *p* < 0.001) in cells lacking Kindlin-2. p21 was also significantly upregulated (4-fold increase, *p* < 0.001) in the K2-KO cells (Fig. 1a). Our focus on SerpinB2 and p21 genes stemmed from recent studies associating these two genes with cancer senescence^16^, and, therefore, to question the role of Kindlin-2 in cancer senescence. Heat map analysis, in fact, identified SerpinB2 as the second most upregulated gene in the K2-KO cells (Fig. 1b). Western blot (Fig. 1c) and qt-RT-PCR (Fig. 1d) analyses, using clones other than the ones used for RNAseq confirmed the upregulation of SerpinB2 in the K2-KO cells compared to their control scram counterparts. Increased expression levels of p21 in the K2-KO cells was also confirmed by western blot analysis (Fig. 1e).

Senescence can be induced by serial passage of cultured cells^18–20^. To further investigate the potential role of Kindlin-2 in senescence through the induction of SerpinB2 and p21, we compared expression levels of SerpinB2 and p21 in young control (Scram) or K2-KO MDA-MB-231 cells (less than 3 passages) and same cells that were continually passaged for at least 16 times (P16). As control for senescence, cells were treated with 1 mM JQ1 for a week, a small molecule targeting bromodomains of BET proteins^21,22^ that also induces irreversible senescence of cultured cells^23^. Expression levels of SerpinB2 were increased >10-fold in the Passage 16 K2-KO and the senescent cells compared to the Young K2-KO cells (Fig. 1e). p21 was also induced to a similar extent (20-fold increase in the P16 and Senescent cells; Fig. 1e). Additionally, AURK-A expression, which is known to decrease in senescent cells, became almost undetectable in the Pa16 K2-KO cells (Fig. 1e). These results were duplicated in a second human BC cell line, BT549 (Fig. 1f); as well as in the 4T1 murine BC cells (Fig. 1g). Thus, loss of Kindlin-2 regulates senescence by inducing senescence-specific genes in BC cells.

In vivo experiments provided independent support for the inverse relationship between Kindlin-2 and SerpinB2 expression. Immunostaining of tumor xenografts derived from NSG mice (MDA-MB-231 cells) or Balb/C mice (4T1 cells) that were implanted in the mammary fat pads with Kindlin-2-deficient (K2-KO) or either control (Scram) cells^11,14^, showed a >7-fold increase (p < 0.001) in SerpinB2 staining in the tumors derived from MDA-231-K2-KO cells (Fig. 1h, k) as well as in the tumors derived from Kindlin-2-deficient-4T1 cells (Fig. 1i, l) compared to their respective Scram controls. Therefore, both *in vitro* and *in vivo* analyses affirmed the role of Kindlin-2 in the upregulation of SerpinB2.

### *Senescence-associated-β-galactosidase activity is activated in Kindlin-2-deficient BC cells*

**Fig. 2:**
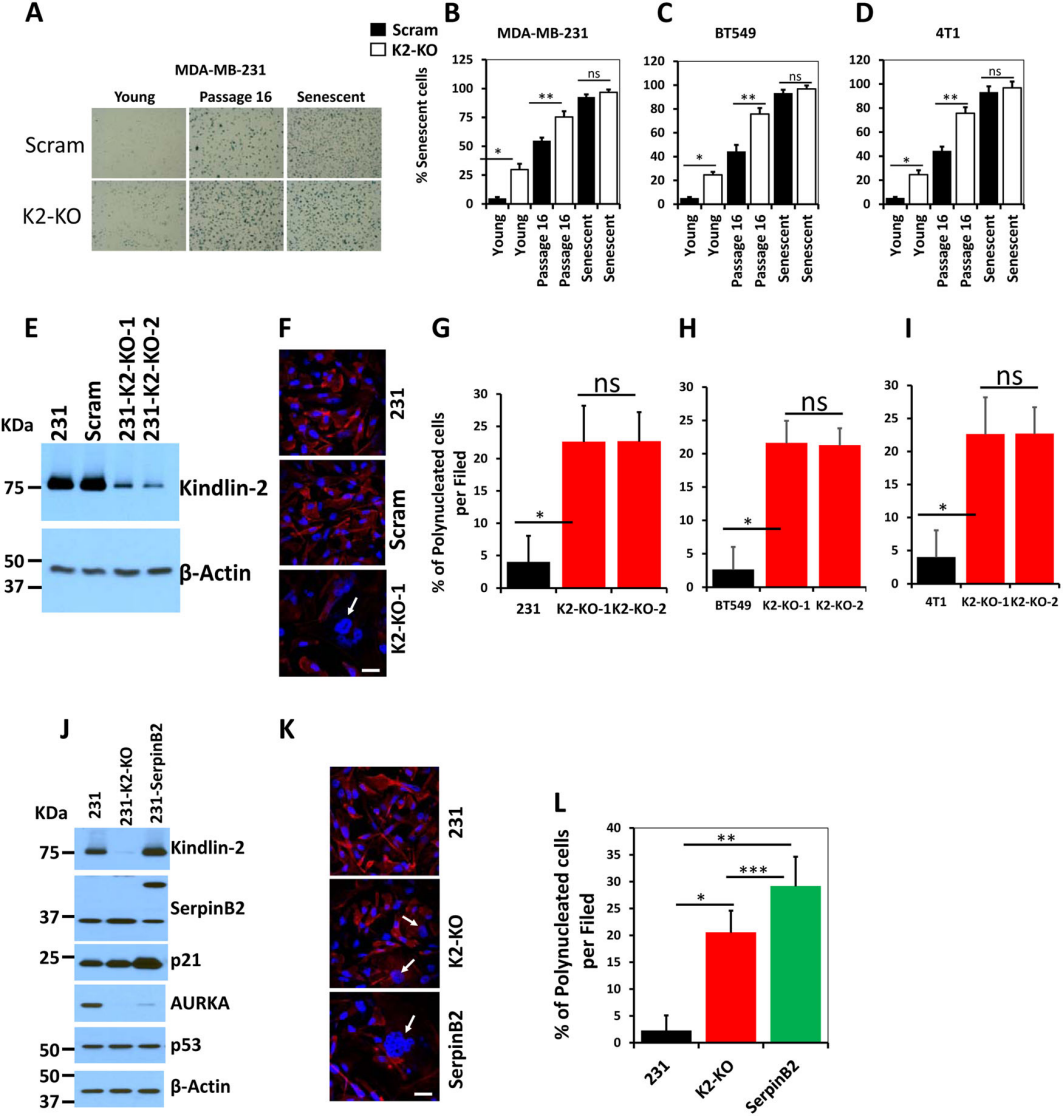
Senescence-associated β-galactosidase activity is activated in Kindlin-2-deficient breast cancer cells, and loss of Kindlin-2 induces polynucleation of BC cells that can be enhanced by overexpression of SerpinB2. **a** Representative micrographs of young, passage 16 and senescent control (scram) or Kindlin-2-deficient (K2-KO) MDA-MB-231 cells that were stained for senescence-associated (SA) β-galactosidase activity (blue staining). **b–d** Quantification of the % of cells of SA-β-galactosidase activity in MDA-MB-231 **b**, BT549 **c**, and 4T1 cells **d**. Data are representative of 3 independent experiments (ns, not significant; *, **p < 0.05, student t-test). **e** Western blots with the indicated antibodies on lysates of parental (231), control (Scram), Kindlin-2-deficient (K2-KO1 and K2-KO-2) cells. Anti-β-Actin (lower panel) was used as a loading control. **f** Representative confocal microscopy micrographs of parental (231, top panel), control (Scram, middle panel) or Kindlin-2-deficient (K2-KO-1, lower panel) MDA-MB-231 cells that were stained for actin filament (red). Nuclei were counterstained with DAPI (blue). White arrows point to polynucleated cells. Scale bar, 20 μm. **g–i** Quantification of the % of polynucleated cells in MDA-MB-231 **g**, BT549 **h**, and 4T1 **i** cells. **j** Western blots with the indicated antibodies on lysates of parental (231), Kindlin-2-deficient (K2-KO) or SerpinB2-overexpressing MDA-MB-231 (231-SepinB2) cells. Anti-β-Actin (lower panel) was used as a loading control. **k** Representative confocal microscopy micrographs of parental (231, top panel), Kindlin-2-deficient (K2-KO, middle panel) or SerpinB2-overexpressing MDA-MB-231 (SepinB2, bottom panel) cells that were stained for actin filament (red). Nuclei were counterstained with DAPI (blue). White arrows point to polynucleated cells. Scale bar, 20 μm. **l** Quantification of the % of polynucleated cells shown in Fig. 3g. Data are representative of 3 independent experiments (*, **, ***p < 0.05, student t-test)

One of the hallmarks of senescence is increased activity of the senescence-associated-(SA)-β-galactosidase. Control (Scram) or Kindlin-2-deficient MDA-MB-231, BT549 and 4T1 BC cells were cultured for a maximum of 3 passages (Young) or for at least 16 passages (P16), and assessed for SA-β-galactosidase activity. As a positive control for senescence induction, cells were treated with JQ1 (1 mM) for ten days. Loss of Kindlin-2 expression (K2-KO) in all three-low passage (Young) cell lines; MDA-MB-231 (Fig. 2a, b), BT549 (Figs. 2c) and 4T1 (Fig. 2d), reached at least 20% (p < 0.05) staining for SA-β-galactosidase activity. Continuous culture (P16) of the control (Scram) cells resulted in an increase of ~50% of senescent cells and more than 80% of the P16 K2-KO cells were positive for SA-β-galactosidase staining (Fig. 2a, b for MDA-MB-231; Fig. 2c for BT549 and Fig. 2d for 4T1 cells). These data indicate that loss of Kindlin-2 expression induced senescence even in young cells. While continuous passaging of the control (P16 Scram) cells significantly (~50%, p < 0.05) increased the number of senescent cells, which remained significantly lower than that obtained in the P16 K2-KO cells. Treatment of cells with JQ1 resulted in staining of almost 100% of cells for SA-β-galactosidase regardless of Kindlin-2 expression.

### *Loss of Kindlin-2 induces polynucleation of BC cells that can be enhanced by overexpression of SerpinB2*

The presence of multiple nuclei within a single cell (polynucleation) is another hallmark of senescence^24^. To investigate whether loss of Kindlin-2 was sufficient to induce polynucleation, Kindlin-2-deficient or Scram MDA-MB-231 cells were allowed to adhere onto glass coverslips and stained for actin. Quantification of the number of nuclei per cell showed that >20% (p < 0.05) of the K2-KO cells (Fig. 2e) had more than one nucleus, compared to less than 5% of polynucleated cells in the parental or the Scram controls (Fig. 2f, g). These findings were also replicated in the BT549 (Fig. 2h) and the 4T1 (Fig. 2i) cells. Importantly, over expression of SerpinB2 (231-SerpinB2, Fig. 2j) in MDA-MB-231 cells that express less SerpinB2 than the Kindlin-2-deficient cells, mimicked the effect of loss of Kindlin-2. It did so by activating p21 expression, inhibiting AURK-A, without affecting expression of p53, which is known to regulate SepinB2 and p21 expression (Fig. 2j). Loss of Kindlin-2 expression was also sufficient to increase cellular polynucleation (Fig. 2k, l). These data suggest that SerpinB2 and p21 are regulated downstream of Kindlin-2 to modulate senescence.

### *Kindlin-2 is required for the orderly progression of the cell cycle*

**Fig. 3:**
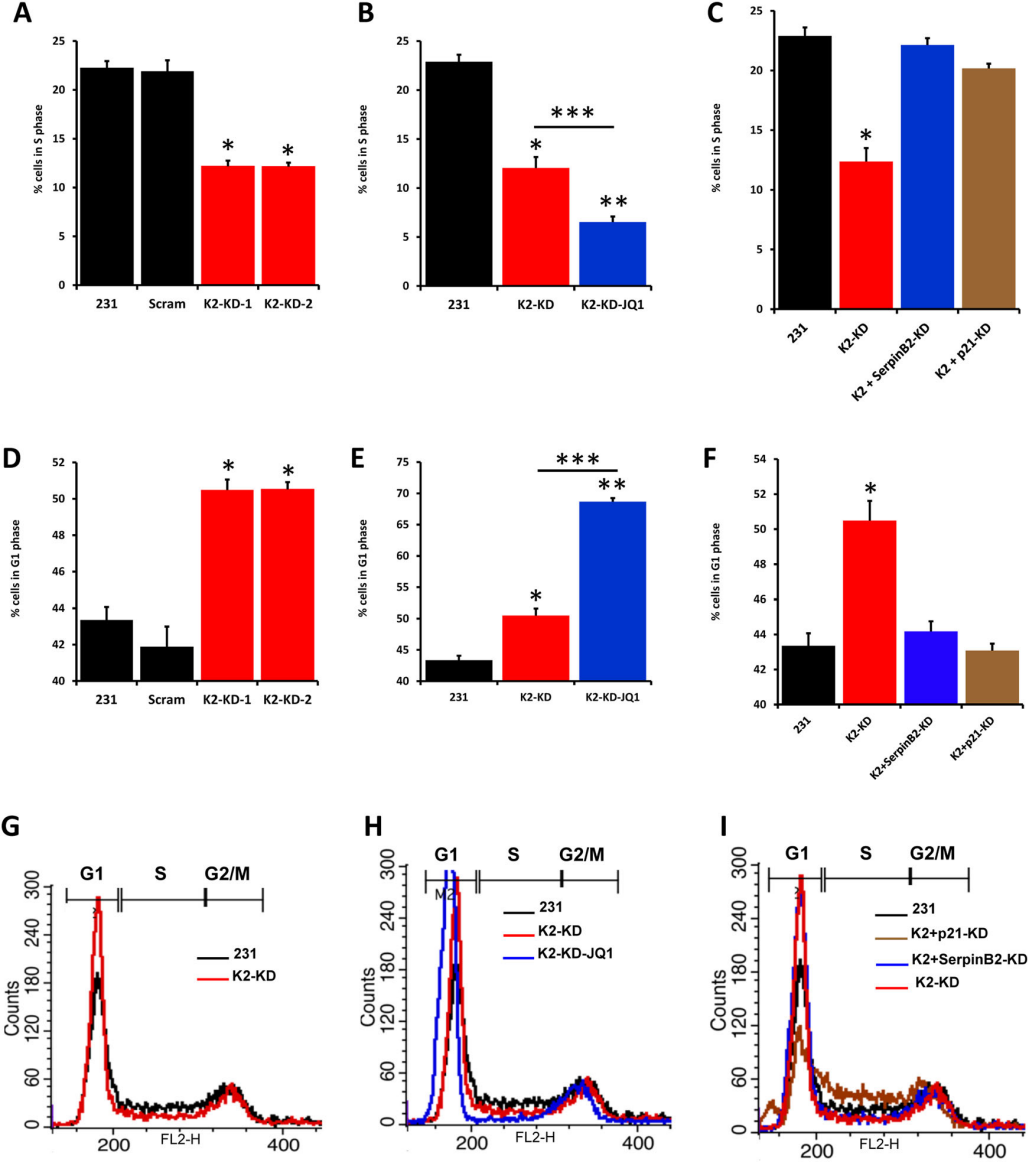
Kindlin-2 is required for the orderly progression of the cell cycle. **a** Quantification of percent of cells in S phase from parental (231), control shRNA(Scram) or Kindlin-2-knockdown MDA-MB-231 cells using K2-shRNA clone 1 (K2-KD-1) or K2-shRNA clone 2 (K2-KD-2). **b** Quantification of percent of cells in S phase from parental (231), Kindlin-2-knockdown MDA-MB-231 cells using K2-shRNA clone 1 (K2-KD-1), or K2-KD MDA-MB-231 cells treated with 1 mM JQ1. **c** Quantification of percent of cells in S phase from parental (231), Kindlin-2-knockdown MDA-MB-231 cells using K2-shRNA clone 1 (K2-KD-1), SerpinB2-Knockdown (SerpinB2-KD) or p21-knockdown (p21-KD). SerpinB2-Knockdown and p21-knockdown were performed in K2-deficient MDA-MB-231 (231-K2-KD). **d** Quantification of percent of cells in G1 phase from parental (231), control (Scram) or Kindlin-2-knockdown MDA-MB-231 cells using K2-shRNA clone 1 (K2-KD-1) or K2-shRNA clone 2 (K2-KD-2). **e** Quantification of percent of cells in G1 phase from parental (231), Kindlin-2-knockdown MDA-MB-231 cells using K2-shRNA clone 1 (K2-KD-1), or K2-KD MDA-MB-231 cells treated with 1 mM JQ1. **f** Quantification of percent of cells in G1 phase from parental (231), Kindlin-2-knockdown MDA-MB-231 cells using K2-shRNA clone 1 (K2-KD-1), SerpinB2-Knockdown (SerpinB2-KD) or p21-knockdown (p21-KD). Data are representative of 3 independent experiments (*, **, ***p < 0.05, student t-test). **g–i** Representative histograms using flow cytometry of parental (231), control shRNA (Scram) or the indicated gene knockdowns and treatments

Cellular polynucleation can be induced by dysregulation of the cell cycle^25^. Specifically, a cell that is unable to transition from G1/S to the G2/M phase will continue to accumulate several nuclei, which triggers a mitotic crisis leading to senescence and subsequent cell death^25^. To determine whether Kindlin-2 expression is required for the proper progression of cell cycle, we quantified the different phases of cell cycle in control (Scram) MDA-MB-231 cells and their Kindlin-2-, SerpinB2- and p21-deficient derivatives. For uniformity of approach, we used shRNA-mediated knockdown of each of the three genes along with scrambled shRNA as a negative control. SerpinB2 and p21 shRNA knockdown were performed in K2-deficient MDA-MB-231 cells, and their selective and efficient knockdown was confirmed by WB (Sup Fig. 1). Knockdown of Kindlin-2 (K2-KD) resulted in ~2-fold decrease (from more than 20 to ~10%, p < 0.05) in the number of cells in the S phase (Fig. 3a, g). Treatment of the Kindlin-2-deficient cells with JQ1 further decreased the number of cells in S phase from ~10% to less than 5% (Fig. 3b, h). Interestingly, the number cells in S phase was restored to almost normal levels when expression of either SerpinB2 or p21 was inhibited in the K2-KD cells (Fig. 3c, i). The opposite effect was observed for G1 phase: loss of Kindlin-2 resulted in a significant (p < 0.05) increase in the number of cells in G1 phase in K2-KD cells (Fig. 3d, g), and it was further increased in these cells by JQ1 treatment (Fig. 3e, h). In addition, the number of cells in G1 phase was restored to normal levels in SerpinB2-KD and the p21-KD in K2-KD cells (Fig. 3f, i). These data not only confirm the involvement of Kindlin-2 in the progression of the cell cycle but also show that the Kindlin-2-mediated modulation of cell cycle progression depends on its regulation of SerpinB2 and p21.

### *Kindlin-2 interacts with p53 in both the cytoplasm and inside the nucleus*

**Fig. 4:**
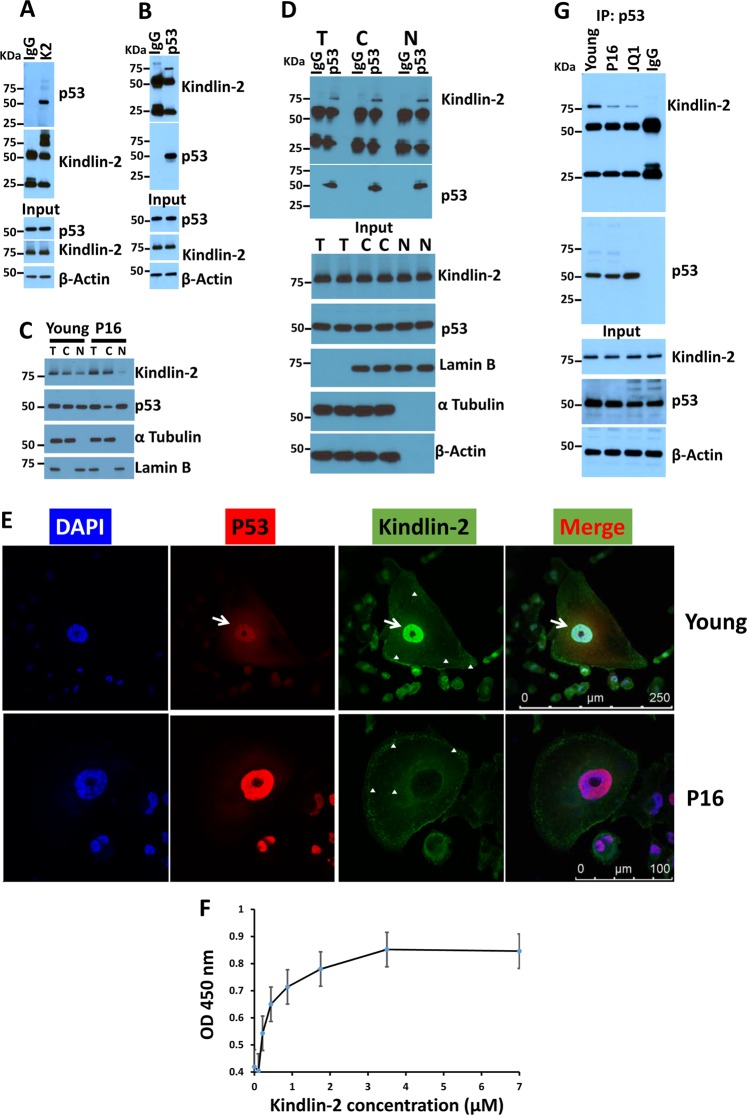
Kindlin-2 interacts with p53 in both the cytoplasm and the nucleus of cancer cells. **a**, **b** Protein lysates prepared from early passage (young) MDA-MB-231 cells was used for immunoprecipitation with mouse anti-Kindlin-2 antibody or control mouse IgG **a**; and mouse anti-p53 antibody or control mouse IgG **b** and subjected to immunoblotting analysis with rabbit anti-p53 antibody (**a**, upper panel) or mouse anti-Kindlin-2 antibody (**b**, upper panel). In control blots, cell lysates were also immunoblotted with mouse anti-Kindlin-2 and rabbit anti-p53 antibodies to show the presence of equal amounts of these proteins in the cell lysates (input panels). β-Actin is a loading control. **c** Total, nuclear and cytoplasmic fractions of protein lysates from early passage (young) and senescent (P16) MDA-MB-231 cells were subjected to immunoblotting with the indicated antibodies. **d** Total, nuclear and cytoplasmic fractions of protein lysates from early passage (young) MDA-MB-231 cells were used for immunoprecipitation with mouse anti-p53 antibody or control mouse IgG, and subjected to immunoblotting analysis with mouse anti-Kindlin-2 antibody (upper panel) or rabbit anti-p53 antibody (upper panel). In control blots (input panels), protein lysates fractions were also immunoblotted with mouse anti-Kindlin-2 and rabbit anti-p53 antibodies to show the presence of equal amounts of these proteins in the lysates as well as the specificity for the nuclear (Lamin B) and the cytoplasmic (α Tubulin). β-Actin is a loading control. **e** Confocal microscopy images of immunofluorescence staining of early passage (young) or senescent (P16) MDA-MB-231 cells that were stained for p53 (Red) or Kindlin-2 (Green). Nuclei were counterstained with DAPI. White arrows point to the co-localization of Kindlin-2 and p53 in the nucleus of young but not senescent cells in the merged image. The white arrowheads point to the localization of Kindlin-2 to focal adhesions in both young and senescent cells. Higher magnifications of other representative images are shown in Supplemental Fig. 2. **f** Interaction of Kindlin-2-GST with purified p53. The binding isotherms of increasing concentrations of GST-tagged WT Kindlin-2 to the wells of microtiter plates coated with p53. The data are expressed as means ± SEM of triplicates of two representative experiments. **g** Protein lysates prepared from early passage (young), passage 16 (P16) or JQ-1 treated (1 mM for 2 days) MDA-MB-231 cells was used for immunoprecipitation with mouse anti-p53 antibody or control mouse IgG and subjected to immunoblotting analysis with rabbit anti-Kindlin-2 antibody or rabbit anti-p53 antibody (upper panels). In control blots (input panels), cell lysates were also immunoblotted with mouse anti-Kindlin-2 and rabbit anti-p53 antibodies to show the presence of equal amounts of these proteins in the cell lysates. β-Actin is a loading control

p21 is a known p53-responsive gene; p53 binds to the p21 promoter and regulates its expression^26^. Recent literature has established that SerpinB2 also is a p53-responsive gene^16^. In fact, both SerpinB2 and p21 were found to be regulated by p53 during cancer cell senescence^16^. To investigate the molecular mechanism whereby Kindlin-2 regulates the expression of SerpinB2 and p21 to induce senescence, we hypothesized that Kindlin-2 may regulate expression of p53-responsive genes through its binding to p53 and restricting its access to the SerpinB2 and p21 promoters. To investigate this possibility, we first demonstrated that both Kindlin-2 and p53 can co-immunoprecipitate in MDA-MB-231 cell extracts (Fig. 4a, b). p53 was detected in the immuno-complexes that were immunoprecipitated with Kindlin-2 (K2) antibody, but not with IgG control (Fig. 4a). The reverse co-immunoprecipitation showed that Kindlin-2 was detected in the immuno-complexes that were immunoprecipitated with p53 antibody, but not with control IgG (Fig. 4b). We also showed that Kindlin-2, along with p53, can be found in both the nuclear and cytosolic fractions of cell lysates of low passage (Young) MDA-MB-231 (Fig. 4c). In senescent (P16) cells, however, the levels of nuclear Kindlin-2 were reduced, while those of p53 were increased (Fig. 4c). The presence of Kindlin-2 and p53 in the same immunocomplexes in both the cytoplasmic and nuclear fractions of young MDA-MB-231 cells was also confirmed in co-immunoprecipitation assays (Fig. 4d). We also used immunofluorescence and confocal microscopy to confirm such co-localization (Fig. 4e). In the young MDA-MB-231 cells, Kindlin-2 and p53 co-localize both in the cytoplasm and the nucleus (Fig. 4e, upper panels and Sup Fig. 2), although nuclear co-localization is much more prominent (white arrows). In the senescent (P16) cells, however, the Kindlin-2/p53 co-localization is significantly diminished; most of Kindlin-2 is restricted to the focal adhesions while most of p53 is localized to the nucleus (Fig. 4e, lower panels and Sup Figs. 2&3). Direct interaction between p53 and GST‐tagged Kindlin-2 was explored using solid phase binding assay with anti-GST-HRP as a dislodging antibody. Full-length Kindlin-2 interacted with immobilized p53 in a concentration‐dependent, saturable manner (Fig. 4f). Using Sigma Plot software, half-maximal binding of Kindlin-2 to p53 occurred at an input Kindlin-2 concentration of 0.48 ± 0.054 µM (means ± SEMs; *n* = 6). The GST binding to p53, which was negligible and did not exceed 10% of total Kindlin-2 binding, was subtracted for normalization. Finally, we found that Kindlin-2/p53 interaction is diminished in the senescent cells as confirmed by co-immunoprecipitation analyses, where we found a significant decrease in the amount of p53 that co-immunoprecipitated with Kindlin-2 in the senescent (P16 and JQ1) cells, compared to the young cells (Fig. 4g). Thus, we confirmed the interaction between Kindlin-2 and p53 in both the nucleus and the cytoplasm of cells, and that this interaction is diminished during senescence.

### *Kindlin-2 binds to p53 to regulate expression of SerpinB2 and p21 at the promoter level*

**Fig. 5:**
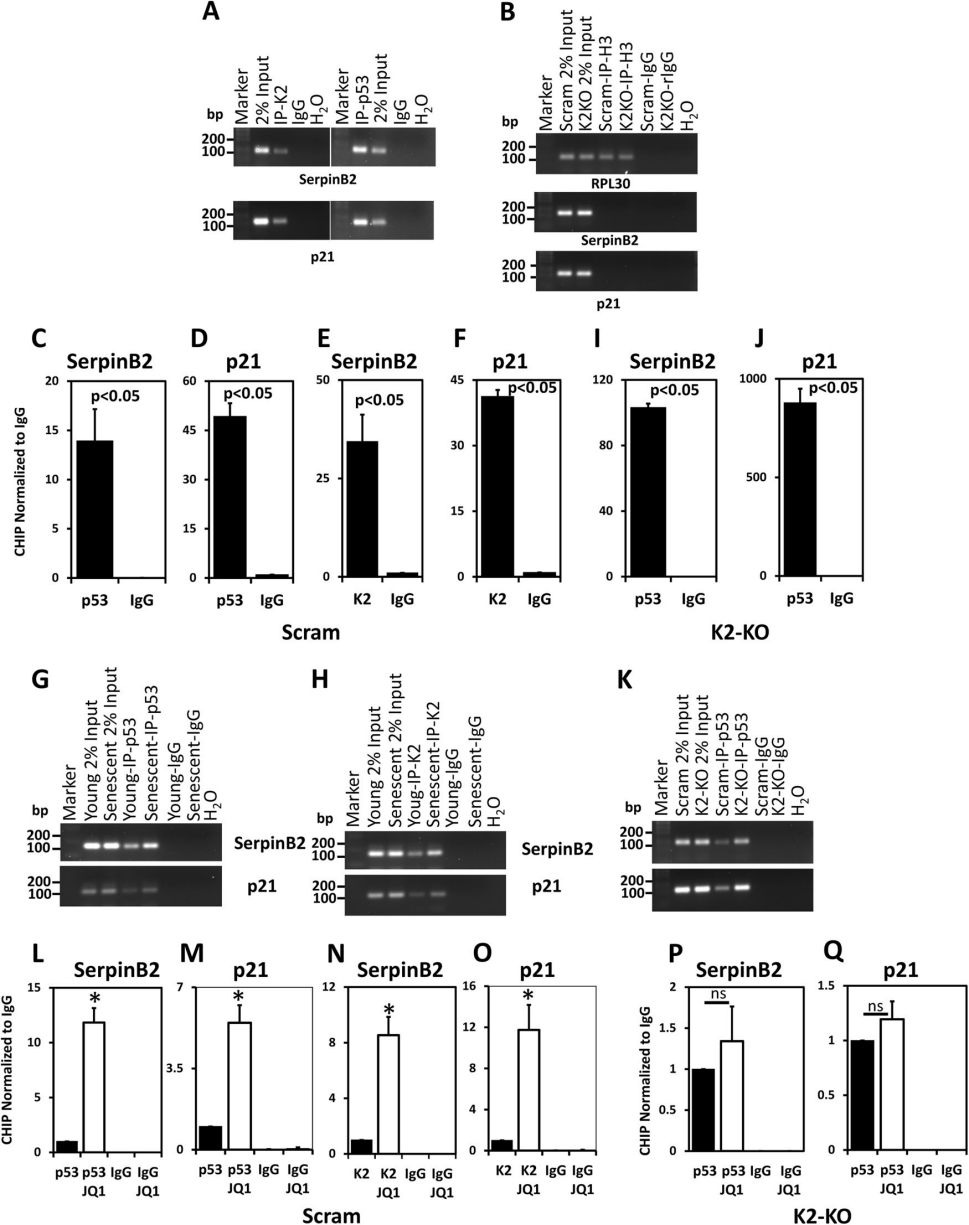
Kindlin-2 binds to p53 to regulate expression of SerpinB2 and p21 at the promoter level. **a** Semi-quantitative agarose gel PCR of CHIP of MDA-MD-231 cells using either anti-Kindlin-2 (left panels) or anti-p53 (right panels) followed by PCR amplification of SepinB2 promoter (upper panel) or p21 promoter (lower panels). The corresponding IgG was used as a negative control for the immunoprecipitation and 2% input of the DNA-chromatin complex was used as a positive control for the PCR. **b** Control CHIP and PCR experiment using anti-Histone H3 for immunoprecipitation of chromatin-DNA complex from control (Scram) or Kindlin-2-deficient (K2-KO) MDA-MB-231 cells, followed by Semi-quantitative agarose gel PCR for the promoter of RPL30 (upper panel), SerpinB2 (middle panel) or p21 (lower panel). Only the RPL30 promoter was amplified in the H3-immunoprecipitates. IgG was used as a negative control for the immunoprecipitation and 2% input of the DNA-chromatin complex was used as a positive control for the PCR. **c–f** Quantitative real-time PCR of DAN-chromatin immunoprecipitates of control (Scram) MDA-MB-231 cells using anti-p53 **c**, **f** or anti-Kindlin-2 **e**, **f**, followed by PCR of SepinB2 **c,** **e** and p21 **d**. **f** promoter, and normalized to the CHIP PCR of the corresponding IgG. **i**, **j** Quantitative real-time PCR of DNA-chromatin immunoprecipitates of Kindlin-2-deficient (K2-KO) MDA-MB-231 cells using anti-p53, followed by PCR of SepinB2 promoter **i** and p21 promoter **j**, and normalized to the CHIP PCR of the corresponding IgG. **g–k** Semi-quantitative agarose gel PCR CHIP of young or senescent MDA-MD-231 cells using either anti-p53 **g** or anti-Kindlin-2 **h**, or Kindlin-2-deficient MDA-MB-231 cells using anti-p53 **k**, followed by PCR amplification of SepinB2 promoter (upper panels) or p21 promoter (lower panels). The corresponding IgG was used as a negative control for the immunoprecipitation and 2% input of the DNA-chromatin complex was used as a positive control for the PCR. **l–o** Quantitative real-time PCR of DNA-chromatin immunoprecipitates of untreated or JQ1-treated control (Scram) MDA-MB-231 cells using anti-p53 **l**, **m** or anti-Kindlin-2 **n**, **o**, followed by PCR of SepinB2 promoter **l**, **n** and p21 promoter **m**, **o**, and normalized to the CHIP PCR of the corresponding IgG. (**p** and **q**) Quantitative real-time PCR of DNA-chromatin immunoprecipitates of untreated or JQ1-treated Kindlin-2-deficient (K2-KO) MDA-MB-231 cells using anti-p53, followed by PCR of SepinB2 promoter **p** and p21 promoter **q**, and normalized to the CHIP PCR of the corresponding IgG. Data are representative of 3 independent experiments (*p < 0.05, student *t*-test)

Next, we used chromatin immunoprecipitation assays to show that both Kindlin-2 (Fig. 5a, left panels) and p53 (Fig. 5a, right panels) bind to SerpinB2 (Fig. 5a, upper panels) and p21 (Fig. AC, lower panels) promoters. Histone H3 and its target gene RPL30 were used as a control (Fig. 5b, upper panel), for the specificity of the assay: neither SerpinB2 (Fig. 5b, middle panel), nor p21 (Fig. 5b, lower panel) promoters were amplified in the chromatin immunoprecipitates that were generated using the Histone H3 antibody. The binding of p53 and Kindlin-2 to the promoters of SerpinB2 and p21 were further confirmed by quantitative PCR of the chromatin immunoprecipitates (Fig. 5c-f). Binding of p53 to SerpinB2 (Fig. 5c, g, upper panel) and p21 promoter (Fig. 5d, g, lower panel) was ~15-and ~50-fold more efficient, respectively, than to control IgG. Similarly, binding of Kindlin-2 to the SerpinB2 promoter (Fig. 5e, h, upper panel) and p21 promoter (Fig. 5f, h, lower panel) was ~35- and ~45-fold more efficient, respectively, than to control IgG. Binding of either p53 or Kindlin-2 to the promoters of SerpinB2 and p21 were further increased in the senescent cells compared to their young counterparts (Fig. 5g, h). Furthermore, we found that, in the absence of Kindlin-2 (K2-KO), the binding of p53 to the promoter of SerpinB2 (Fig. 5i, k) was increased by ~8–15-fold in the Scram cells (Fig. 5c) compared to ~120-fold in K2-KO cells (Fig. 5i). Similarly, we found that the binding of p53 to the p21 promoter (Fig. 5j, k) was increased by only ~20–50-fold in the scram cells (Fig. D) compared to ~1000 in K2-KO cells (Fig. 5j, k). Here again, in the absence of Kindlin-2 (K2-KO), p53 was able to bind more efficiently to the respective promoters of SerpinB2 (Fig. 5k, upper panel) and p21 (Fig. 5k, lower panel) in the senescent cells compared to their young counterparts, as judged by the increase of the PCR signal in the chromatin immunoprecipitates from the senescent cells. Finally, treatment of control (Scram) MDA-MB-231 cells with senescence inducing agent JQ1 increased the binding of p53 to the SerpinB2 (Fig. 5l) and p21 (Fig. 5m) promoters, compared to the untreated Scram cells. JQ1 had a similar effect on the binding of Kindlin-2 to the promoters of SerpinB2 (Fig. 5n) and p21 (Fig. 5o) in the control (Scram) cells. However, in the Kindlin-2-deficient (K2-KO) cells, treatment with JQ1 did not have any significant added effect on the binding of p53 to the promoter of either SerpinB2 (Fig. 5p) or p21 (Fig. 5q), suggesting that the interaction between Kindlin-2 and p53 and their binding to the promoters of SerpinB2 and p53 may not depend on JQ1. Together, our data shows that Kindlin-2 negatively regulates the activity of p53 by restricting its binding to the p53-responsive genes and activating their expression to induce senescence.

### *The Kindlin-2-mediated regulation of senescence may not depend on its binding to integrin or actin*

**Fig. 6:**
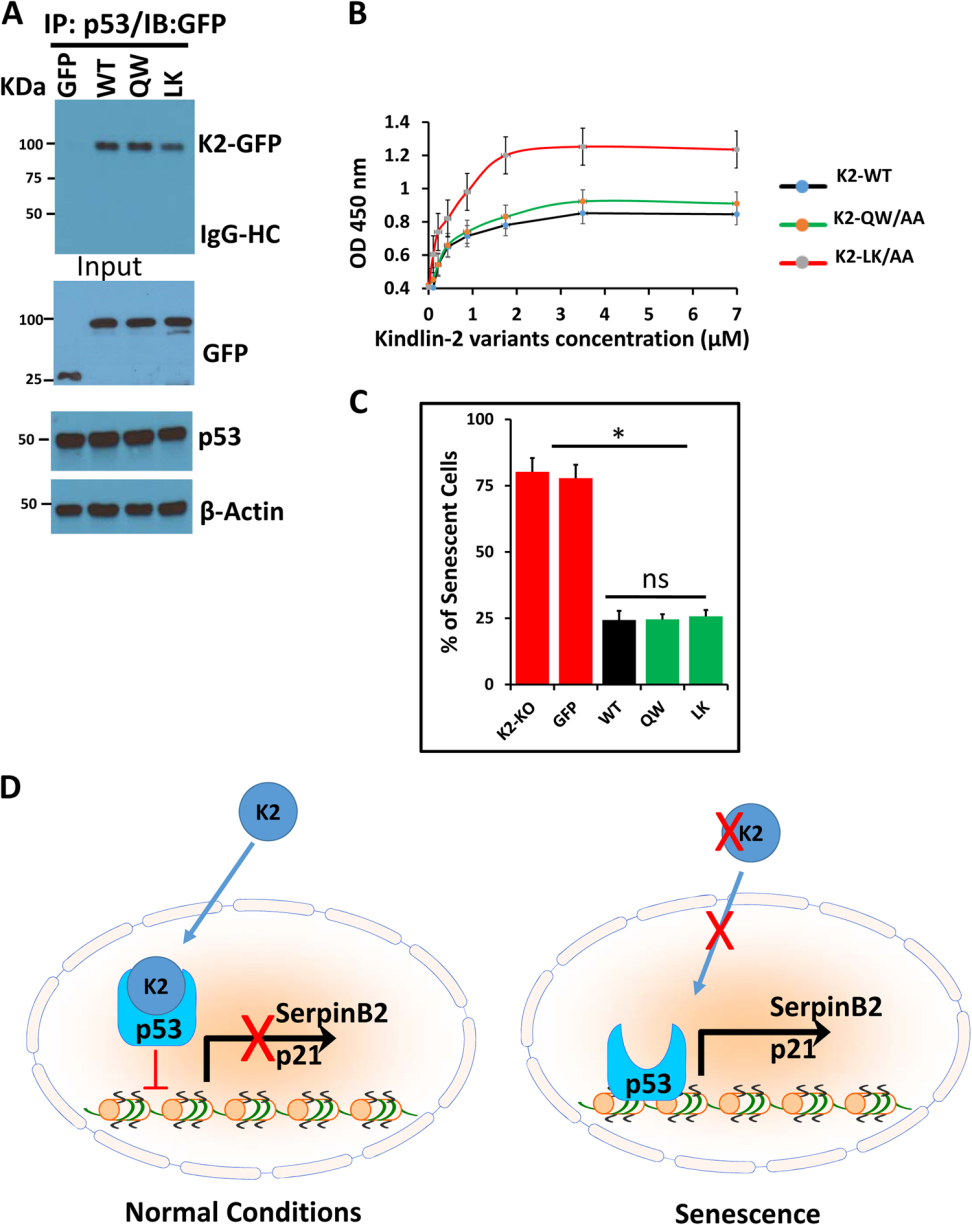
The Kindlin-2-mediated regulation of senescence may not depend on its binding to integrin or actin **a** Kindlin-2 deficient (K2-KO) MDA-MB-231cells were transiently transfected with expression vectors for control GFP, wild-type Kindlin-2, QW/AA-Kindlin-2 or LK/AA-Kindlin-2 mutants fused to GFP. The corresponding total protein lysates were used for immunoprecipitation with rabbit anti-p53 antibody, and subjected to immunoblotting analysis with mouse anti-GFP antibody (upper panel). In control blots (Input blots), the same cell lysates were also immunoblotted with mouse anti-GFP and rabbit anti-p53 antibodies to show the presence of equal amounts of these proteins in the cell lysates. β-Actin is a loading control. **b** Interaction of Kindlin-2-GST with purified p53. The binding isotherms of increasing concentrations of GST-tagged WT Kindlin-2 and its mutant variants Kindlin-2 (QW/AA) and Kindlin-2 (LK/AA) to the wells of microtiter plates coated with p53. The data are expressed as means ± SEM of triplicates of two representative experiments. **c** Quantification of SA β-galactosidase staining in the Kindlin-2-deficient (K2-KO) MDA-MB-23 and the K2-KO cells transfected with the control GFP, wild type Keindlin-2 (WT), QW/AA (QW) or LK/AA (LK) Kindlin-2 mutants. Data are representative of 3 independent experiments (*p < 0.05, student *t*-test). **d** Model describing the role of Kindlin-2 in the regulation of senescence in breast cancer

Several studies have described the requirement of Kindlin-2 for the activation of integrins depends on its binding to the cytoplasmic tails of the beta subunit of integrins^9^. Mutation of the amino acid doublet, K2(QW^615^) in the C-terminal F3 subdomain of Kindlin-2 was found to impair the Kindlin-2/integrin interaction and to severely affect the inside-out signaling of integrins^27^. More recently, we also found the Kindlin-2/Actin interaction centered at K2(LK^47^) in the F0 subdomain impaired Kindlin-2 mediated actin bundling^28^. We, therefore, asked whether Kindlin-2-mediated regulation of senescence is dependent on its binding to integrin and/or actin. We transiently transfected Kindlin-2-deficient (K2-KO) MDA-MB-231 cells with either control GFP, wild-type Kindlin-2- (K2-WT), K2(QW^615^/AA)- or K2(LK^47^/AA)-GFP fusion constructs and the resulting protein lysates were immunoprecipitated with anti-p53 antibody, followed by immunoblotting with anti-GFP antibody (Fig. 6a). p53 was found in the same protein immunocomplexes containing either wild-type Kindlin-2, the QW/AA or LK/AA Kindlin-2 mutants. Interestingly, no significant difference in binding levels was observed between p53 and the Kindlin-2 mutants, compared to wild-type Kindlin-2, as judged by the intensity of the K2-GFP bands in the IP blot (Fig. 6a). We also used ELISA binding assays to show that mutation of the integrin binding site (K2QW/AA) and to the actin binding site (K2LK/AA) did not affect the direct binding of Kindlin-2 to p53 (Fig. 6b). Finally, we assessed the effect of these two Kindlin-2 mutants on senescence in the K2-KO cells by means of SA-β-galactosidase assay (Fig. 6c). SA-β-galactosidase-positive cells were not significantly different between the K2-KO cells and the K2-KO cells expressing control GFP. Re-expression of wild-type Kindlin-2 (WT) reduced the number of senescent cells by almost 4-fold (p < 0.05), which is consistent with the anti-senescence activity of Kindlin-2. More importantly, both the Kindlin-2 QW/AA and LK/AA had similar effect as did wild-type Kindlin-2; they all resulted in ~4-fold reduction in the number of senescent cells, suggesting that the Kindlin-2/integrin and Kindlin-2/actin interaction may have no major effect on Kindlin-2 mediated regulation of cell senescence.

## Discussion

This study demonstrates that loss of Kindlin-2 inhibits BC tumor growth in part by regulating cellular senescence. Nuclear function of Kindlin-2 was previously reported to form a transcriptional complex with β-catenin to regulate Wnt signaling in BC^29^. Our study identifies a previously undocumented function of Kindlin-2 in the nucleus that depends on its interaction with p53 to regulate expression of p53-responsive genes. As a consequence of this interaction, Kindlin-2 is involved in inducing senescence. Senescence was first described by Hayflick and Moorhead^1^ as a stress-induced, durable cell-cycle arrest of replication-competent cells. Since then, senescence has become widely recognized as a powerful cellular mechanism for the regulation of cell growth in normal physiological and pathological settings, including cancer^4^. Among established hallmarks of senescence are sustainable growth arrest, increased expression of p21 and SA-β-galactosidase activity, and cell cycle arrest^4^. In the present study, we demonstrate that these markers of senescence are all associated with loss of Kindlin-2 in BC cells. Despite the complexity of the molecular mechanisms that drive senescence, only two well-defined signaling pathways (p53 and pRB) have been established as major regulators of the senescence phenotype^30^. Our study is the first to implicate Kindlin-2 in p53-mediated regulation of senescence. We applied a combination of molecular, genetic and pharmacologic manipulations, as well as different biochemical and cell imaging analyses *in vitro* and mouse models, to investigate the role of Kindlin-2 in modulating the p53-mediated regulation of senescence in BC. We showed that loss of Kindlin-2 in BC cell lines of both human and mouse origin resulted in a significant increase in expression levels of SerpinB2 and p21, the two well-established p53-responsive genes, both *in vitro* and in tumor xenografts. As a consequence, several hallmarks of senescence were activated, including (*i*) increased SA-β galactosidase activity, (*ii*) a significant increase in the number of polynucleated cells, and (*iii*) induction of cell cycle arrest. Mechanistically, we demonstrated that Kindlin-2 physically interacts with p53 and this interaction prevents the binding of p53 to the promoters of SerpinB2 and p21. Loss of expression of Kindlin-2 lifts this inhibitory effect since p53 can now bind to the SerpinB2 and p21 promoters and drive their expression, which in turn leads to activation of the senescence phenotype. Thus, we have established a Kindlin-2/p53/SerpinB2 signaling axis as a key regulator of senescence in BC. It remains to be seen whether Kinldin-2 is also involved in pRB-mediated senescence.

While p21 is a well-established regulator of senescence, very limited information is available with respect to the involvement of SerpinB2 in this context. Recently, Hsieh et al.^16^ showed that SerpinB2 is required for the stabilization of p21 in senescent cells. SerpinB2, also known as PAI2, is a paralog of the plasminogen activator inhibitor-1 (PAI1)^31^. SerpinB2, unlike PAI1, does not have a readily demonstrable anti-fibrinolytic activity. Loss of expression of SerpinB2 was, however, shown to be associated with the activation of tumor growth and metastasis in several cancer types, including BC^32–34^. Expression levels of SerpinB2 was also shown to correlate negatively with survival of patients with lung carcinomas^35^. Also, downregulation of SerpinB2 was found to contribute to chemoresistance in head and neck carcinomas^36^. Interestingly and in accord with the literature, SerpinB2 and Kindlin-2 seem to play opposing roles in cancer:SerpinB2 behaves as a tumor suppressor^32–36^ while Kindlin-2 acts as tumor promoter^9,11,14,15^. Many questions remain to be considered. For example, Kindlin-1 and Kindlin-3, the two other members of the kindlins family, have been linked to cancer pathology, including BC^37,38^. Interestingly, Kindlin-1 was found to regulate senescence in primary keratinocytes derived from patients with Kindler Syndrome^39^. Kindlin-3, on the other hand has not yet been associated with the senescence phenotype. Whether the Kindlin-mediated regulation of senescence involves the same molecular pathway utilized by Kindlin-2 remains to be investigated, keeping in mind that a pathway overlap is more unlikely since members of kindlins family do not compensate for each other, even when present in the same cell background^11,38^. Also, integrins, which require Kindlins for their activation, were also found to regulate senescence^12,13^. Nevertheless, our data suggest that inhibition of Kindlin-2/integrin interaction resulting from mutation of K2(QW) residues did not compromise Kindlin-2 regulation of senescence.

Together, our findings establish a Kindlin-2/p53 signaling axis that leads to regulation of SerpinB2 and p21 expression to induce senescence in cancer cells. We further identify a previously unknown, and somehow surprising function of Kindlin-2 in the nucleus (Fig. 6d). The present study also adds the regulation of senescence to the multitude of functions of Kindlin-2 during the development, progression and metastasis of cancer^11,14,15^, therefore identifying kindlin-2 as a potential target for therapeutic investigations.

## Materials and methods

### Ethics statement

This study used 6- to 8-week-old female NSG or Balb/C mice (Jackson Laboratory, Farmington, CT). All methods were performed in accordance with the guidelines and regulations set and approved by the Cleveland Clinic and NIH.

### Cell Lines and Reagents

BC cell lines were obtained from American Type Culture Collection (ATCC) and maintained according to the manufacturer’s protocols. Cell lines were also routinely authenticated by STR DNA fingerprinting analysis. 4T1 cells were engineered to stably express firefly luciferase by transfection with pNifty-CMV-luciferase and selection (500 μg/ml) with Zeocin (Invitrogen, Carlsbad, CA). Kindlin-2-KO cells were generated by lentiviral transduction as described^11^. We used two independent and verified Kindlin-2-specific sgRNAs for each of the human and mouse Kindlin-2 and a scrambled sgRNA (i.e., nonsilencing sgRNA,^11^). Loss of Kindlin-2 expression was verified by Western blot.

### Antibodies

The following primary antibodies were from Cell Signaling Technology: Rabbit anti α-Tubilin (1:1000), mouse anti-p53 (1:1000) and rabbit anti-p53 (1:1000 for WB and 1:500 for immunofluorescence). Mouse monoclonal anti-Kindlin-2, clone 3A3 (1:2000 for WB and 1:500 for immunofluorescence) was from Millipore; rabbit polyclonal anti SerpinB2 (1:1000) and rabbit polyclonal anti Lamin B1 (1:3000) were from Abcam. Goat horseradish peroxidase-conjugated anti-mouse IgG (1:2000) and goat horseradish peroxidase-conjugated anti-rabbit IgG (1:2000) were from Calbiochem. Mouse monoclonal anti-Actin (1:5000) was from Sigma. Alexa Fluor Plus 594-conjugated anti-Rabbit IgG and Alexa Fluor Plus 488-conjugated anti-mouse IgG were from Invitrogen. Vecta-shield with 4′,6-diamidino-2-phenylindole (DAPI) was from Vector Laboratories. Gel electrophoresis reagents were from Bio-Rad.

### shRNA-mediated knockdown and CRISPR/Cas9 gene editing-mediated targeting

Short guide (sg)RNA sequences and lentivirus CRISPR-mediated KO of Kindlin-2 were performed as described previously^11^. Mixed cell populations resulting from the CRISPR-mediated KO selection were used throughout the study. Lentivirus shRNA clones were purchased from Sigma: Kindlin-2, clone TRCN0000127808 and clone TRCN0000128511; p21, clone TRCN0000307347 and clone TRCN0000294421; SerpinB2, clone TRCN0000052278 and clone TRCN0000379050; non-targeting scrambled control SHC002. The lentivirus-mediated shRNA gene knockdown procedures were also described previously^40^.

### RNA-Seq and biostatistical analyses

Control and K2-KO MDA-MB-231 cells were grown to near confluency (80 to 90%) onto 10-cm tissue culture dishes in DMEM media supplemented with 10% FBS. Passage 16 cells were used for RNA extraction and RNA-Seq analyses.

#### TruSeq Total RNA Stranded Library Preparation and Sequencing

Before beginning any of the library preparations, the RNA for each sample is quality checked using Agilent’s Bioanalyzer 2100 RNA Nano 6000 reagent kit. Each of the samples will ideally have an RNA Integrity Number (RIN) value higher than 8, indicating minimal sample degradation. We used Illumina’s TruSeq Total RNA Stranded Library Preparation Kit to achieve a range from 100 ng to 1 µg of total RNA. Generally, a concentration of 400–500 ng is used per sample, as that concentration falls in the middle of the indicated range. Library preparation is then performed according to the manufacturer’s instructions. Each sample is given a unique index to distinguish it from other samples. The final library size distribution is checked using a Bioanalyzer DNA High Sensitivity assay and if found to be within the correct size range, Qubit is used to determine the concentration of each sample. Samples are then pooled by concentration and an accurate concentration for the pool is determined using the Kapa Library Concentration Kit.

#### Sequencing

Sequencing for up to twelve Total RNA samples is done using Illumina’s HiSeq 2500 set in Rapid Run mode. The sequencing kit used for RNA-Seq is the paired end (PE) 100 cycle kit (meaning that 100 cycles will be used for the forward and reverse directions so a total of 200 cycles is used). This will generate ~30–40 million reads per sample which when used with biological replicates (3 per condition) will yield statistically significant results. Sequencing results are delivered as FASTQ files which can then be aligned and analyzed using bioinformatics.

##### RNA-Seq analysis methods

Sequencing reads generated from the Illumina platform were assessed for quality using FastQC. The reads were trimmed for adapter sequences using TrimGalore. For RNASeq, reads that passed quality control were then aligned to the human reference genome (GRCh38) using the STAR aligner^41^. The alignment for the sequences were guided using the GENCODE annotation for GRCh38. The aligned reads were then analyzed for differential expression using cufflinks^42^, a RNASeq analysis package which reports the fragments per kilobase of exon per million fragments mapped (FPKM) for each gene. The 12 samples were analyzed in 4 groups of 3 (WT, W3, K2, and DKO) and differential expression analysis was performed in a pairwise manner. Differential genes were identified using a significance cutoff of FDR < 0.05. These genes were then subjected to gene set enrichment analysis (GSEA, Broad Institute) to determine any relevant processes that may be differentially over represented for the conditions tested.

### Senescence-associated (SA)-β-galactosidase activity assay

SA-β-galactosidase activity was assessed using Cell Signaling Senescence-associated β-galactosidase Staining Kit (#9860). Briefly, 50,000 cells were seeded onto 6-well plates and allowed to adhere overnight, then washed with PBS and fixed for 15 min in the fixative solution. The fixed cells were then washed twice with PBS and incubated with β-galactosidase staining solution at 37 °C for overnight. For each sample, 15–20 randomly chosen fields were photographed using a camera-equipped bright field microscope (Leica), and at least 300 cells were counted from each field. Data was plotted as the percent of cells with β-galactosidase activity (blue cells).

### Cell cycle

MDA-MB-231 cells were transduced with lentivirus shRNAs described above against Kindlin-2 (K2-KD), SerpinB2 (SerpinB2-KD) or p21 (p21-KD), and subjected to puromycin treatment (5 μg/ml) for 14 days to select for pool of clones with the desired gene knockdown, which was verified by western blot analysis (Supplementary Figure 1). The control and gene knockdown cells seeded onto 6-well tissue culture plates overnight were treated as indicated. Cells were briefly trypsinized and washed once with PBS. Cell cycle analysis was examined using Propidium Iodide Flow cytometry Kit for Cell Cycle Analysis (Abcam) according to manufacturer’s instructions. Briefly, the cells were fixed in 70% ethanol on ice for at least 2 h. The cells were washed with ice-cold PBS and stained in 1× Propidium Iodide /RNase staining solution (5 × 10^5^ cells in 200 μl) for 30 min at 37 °C. Cells were placed on ice and analyzed immediately using a FACS Calibur flow cytometer and the Cell Quest Pro software (BD Biosciences).

### Co-immunoprecipitation and Western blot analyses

Cells were grown in 6-well tissue culture plates until confluency reached ~80%. Cells were then washed twice with ice-cold PBS and lysed on ice with RIPA buffer. The crude lysates were transferred to prechilled tubes and centrifuged at 13K RPM for 15 min at 4 °C. Cleared cell lysates were denatured with SDS sample buffer and resolved (~25 ug) on a 4–20 gradient SDS acrylamide gels (Bio-Rad), followed by transfer to polyvinylidene difluoride membranes (Bio-Rad). Membranes were incubated in 5% bovine serum albumin in PBST buffer for 1 h at room temperature, followed by incubation with the primary antibody (as specified) overnight at 4 °C. Membranes were then washed 3 times with PBST buffer and incubated in the appropriate secondary antibody at room temperature for 1 h, and the signals were developed using the Western Lights chemiluminescence detection kit (PerkinElmer Life Sciences).

For co-immunoprecipitation assays, 500 μg of cellular extracts were incubated with appropriate primary antibodies or normal rabbit/mouse immunoglobin G (IgG) on a rotator at 4 °C overnight, followed by addition of Protein G for 2 h at 4 °C. Beads were then washed four times with RIPA buffer and protease inhibitor mixture. The immune complexes were subjected to SDS-PAGE and immune-blotting as described above.

### Primary tumor growth, bioluminescence imaging and metastasis assays

Parental (scram) or Kindlin-2-deficient MDA-MB-231 cells (10^6^ cells per mouse, *n* = 5) were implanted into the mammary fat pads of female NSG mice. Tumor growth was followed by twice weekly monitoring of tumor volume with digital Vernier calipers. Luciferase-expressing parental (scram) or Kindlin-2-deficient 4T1 cells (10,000 cells per mouse, *n* = 5) were implanted into the mammary fat pads of female Balb/C mice. Tumor growth was quantified using bioluminescence imaging as described previously^11^. The number of mice, as well as the analysis of the resulting tumors, are described in full details in our previously published manuscript^11^.

### ChIP-qPCR analysis and agarose gel electrophoresis

CHIP–quantitative real-time fluorescence PCR (qPCR) was performed using Cell Signaling SimpleChIP Enzymatic Chromatin IP Kit (#9002) per the manufacturer’s instructions. Human p21 and SerpinB2 promoter primers were from Cell Signaling: p21-Forward, 5′-GAGGTCAGCTGCGTTAGAGG-3′; p21-Reverse, 5′-TGCAGAGGATGGATTGTTCA-3′; SerpinB2-Forward, 5′-TCTTGAAACTGGGGCTGACA-3′; SerpinB2-Reverse, 5′-CCTCTGTCTTTTGATCTGTGTCC-3′. These primers have been validated previously (17511890 and 28794016). Antibodies used for ChIP assays were anti-Kindlin-2 (clone 3A3, EMD Millipore) and anti-p53 (clone 7F5, Cell Signaling). The PCR products were resolved on 2% agarose gels, detected with SYBR Safe DNA gel staining (Invitrogen), and documented with Chemidoc MP Imaging Instrument (Bio-Rad).

### Real-time quantitative RT-PCR

TRIzol reagent (Invitrogen, Camarillo, CA) was used to extract total RNA from cancer cell lines according to the manufacturer’s instructions. Quantitative real-time PCR was performed, as described previously^15,38,43^. RT^2^ qPCR Primer Assays for SerpinB2 (Cat. No. PPH00793C) and control GAPDH (Cat. No. PPH00150F) were obtained from Qiagen.

### Immunofluorescence and confocal microscopy

Cells were grown on glass coverslips and fixed in 4% paraformaldehyde for 20 min in PBS at room temperature and washed with PBS. The cells were then permeabilized in 0.2% Triton X-100 in PBS for 15 min, washed again with PBS, and incubated in the blocking solution containing 5% bovine serum albumin (Sigma) in PBS for 2 h at room temperature. Primary as well as secondary antibodies were diluted to the recommended concentration in 5% bovine serum albumin in PBS. Cells were incubated with the primary antibody for 1 h, washed with PBS, and then incubated with the secondary antibodies for 1 h. Actin filaments (F-actin) were stained with rhodamine-conjugated phalloidin (Molecular Probes) in PBS. The coverslips were mounted on object slides using Vectashield mounting medium containing 4′,6-diamidino-2-phenylindole (DAPI, Vector Laboratories). Fluorescence images were captured using a Nikon TE2000-E inverted microscope. Numbers of nuclei per cell were counted in at least 20 different fields using the ImageJ software according to the parameters described in ImageJ user guide (http://rsbweb.nih.gov/ij/docs/guide/146.html). Average values of 5 different images were plotted.

### Expression vectors and transfections

GFP-tagged SerpinB2 plasmid clone HG10641-ACG was purchased from Sino Biological and sequence-verified using a 3100 Genetic Analyzer (ABI Prism). GFP-tagged Kindlin-2 was generated via ligation of the full length wild-type Kindlin-2 (accession number NM_006832) in- frame with GFP in the pEGFP-C2 expression vector (Clontech). Cloning was confirmed by sequencing and western blot. The resulting plasmid construct was used as a template for site-directed mutagenesis to introduce the K2-QW^615^/AA or K2-LK^47^/AA mutations. The GFP-recombinant vector or the empty GFP expression control vector was used for either transient or stable transfections using standard protocols, and the correct size of the fusion proteins were verified by western blot analysis. Oligonucleotide primers used for sequencing were from Qiagen and are available upon request.

### Immunohistochemistry

Tissues were collected at the indicated times, snap-frozen in optimal cutting temperature (OCT) medium (Sakura Finetek, Japan), and 8-μm sections were prepared and stained with the following antibodies; rabbit anti-SerpinB2 (1:100) (Abcam), as described previously^11,15,38^. Stained sections were analyzed using fluorescent or bright-field imaging microscopy (Leica) and ImagePro Plus Capture and Analysis software (Media Cybernetics). SerpinB2 positive areas were quantified in 15 independent fields/section using Image Pro-Plus software^11,38^.

### Solid phase direct binding assays

Full-length Kindlin-2 and its mutant variants K2-QW^615^/AA or K2-LK^47^/AA were expressed in Escherichia coli as GST fusion proteins and purified on Glutathione-Sepharose (GE Healthcare) according to manufacturer’s instructions. p53 protein in TBS buffer (Abcam) was immobilized onto 96-well microtiter plates (Corning Costar Corp., Cambridge, MA) at 5 μg/well for 20 h at 4 °C. After post-coating with 3% BSA for 1 h at 37 °C, Kindlin-2 (WT, QW/AA, LK/AA)-GST-tagged and GST protein were added (0–7 µM) in TBS buffer containing 1 mM CaCl2, 1 mM MgCl2 and incubated for 2 h at 37 °C. After 3 washings with TBS anti-GST-HRP Ab (1:2000) was added (100 µl/well) and incubated for 1 h at RT. The plates were washed 3 times with TBS, developed with 1-Step Ultra TMB-ELISA substrate (ThermoScientific) and absorbance at 450 nm was measured. In binding isotherm studies, input concentrations of the GST–Kindlin-2 variants required for half-maximal binding to immobilized p53 were estimated using the Sigma Plot software (SPSS) in which the data were fitted to a one-site binding equation. GST alone was as a negative control for binding to p53 and for the normalization.

### Statistical analysis

Experiments were done in triplicate and analyzed using the Student’s *t*-test. In calculating two-tailed significance levels for equality of means, equal variances were assumed for the two populations. Results were considered significant at *p* < 0.05.

## Supplementary information

Supplementary Figure 1

Supplementary Figure 2

Supplementary Figure 3

Legends for Supplementary Figures

## Data Availability

The datasets generated and analyzed during this study that are not included in the published article, including the RNASeq raw data, are available from the corresponding author upon reasonable request.
