# Supplementary figures and images for: The Kindlin2-p53-SerpinB2 signaling axis is required for cellular senescence in breast cancer
Source: Cell Death Dis. 2019 Jul 15;10(8):539. doi: 10.1038/s41419-019-1774-z (PMC6629707; doi:10.1038/s41419-019-1774-z)

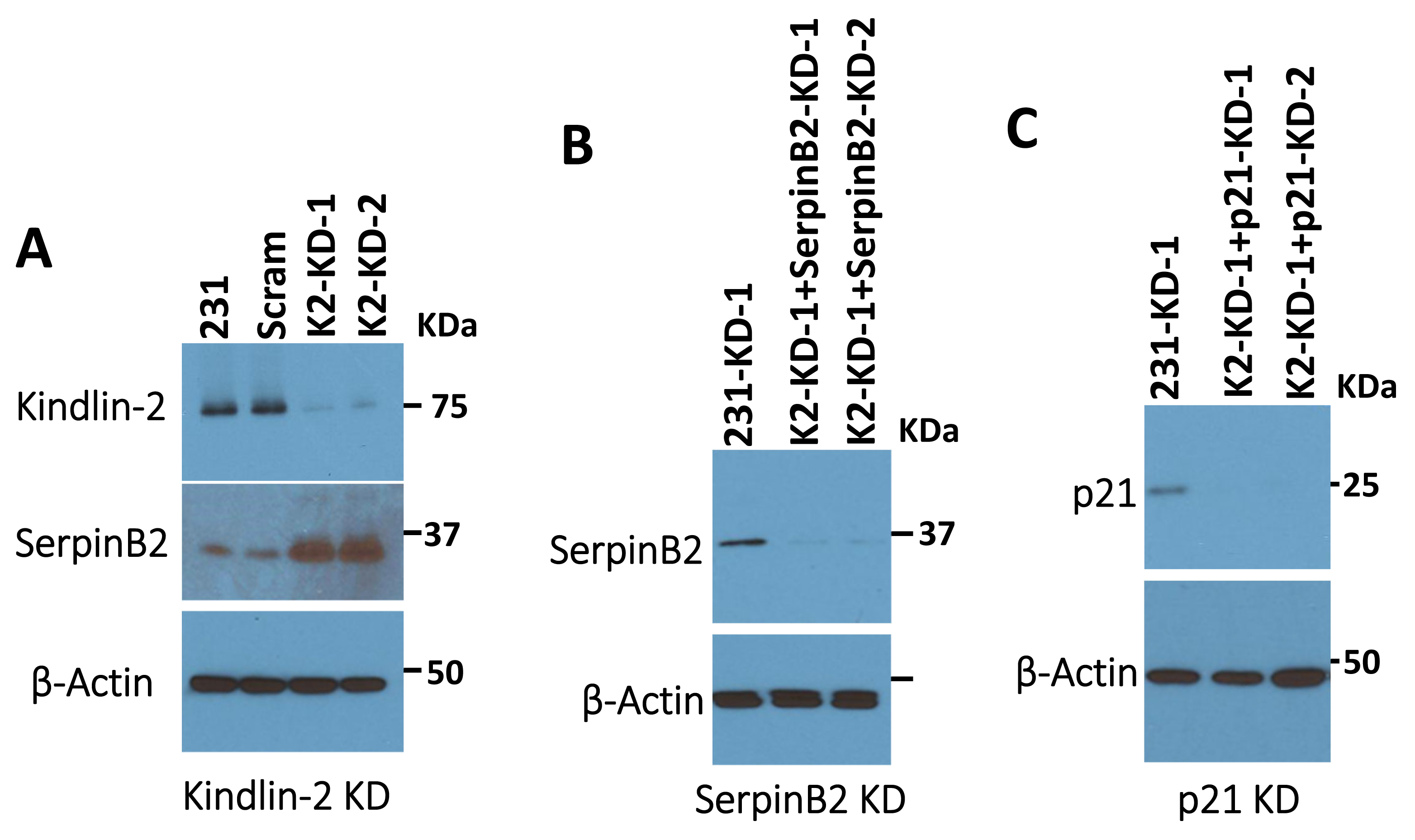

Supplement: Supplementary file 1 — Supplementary Figure 1 [file 41419_2019_1774_MOESM1_ESM.tif]

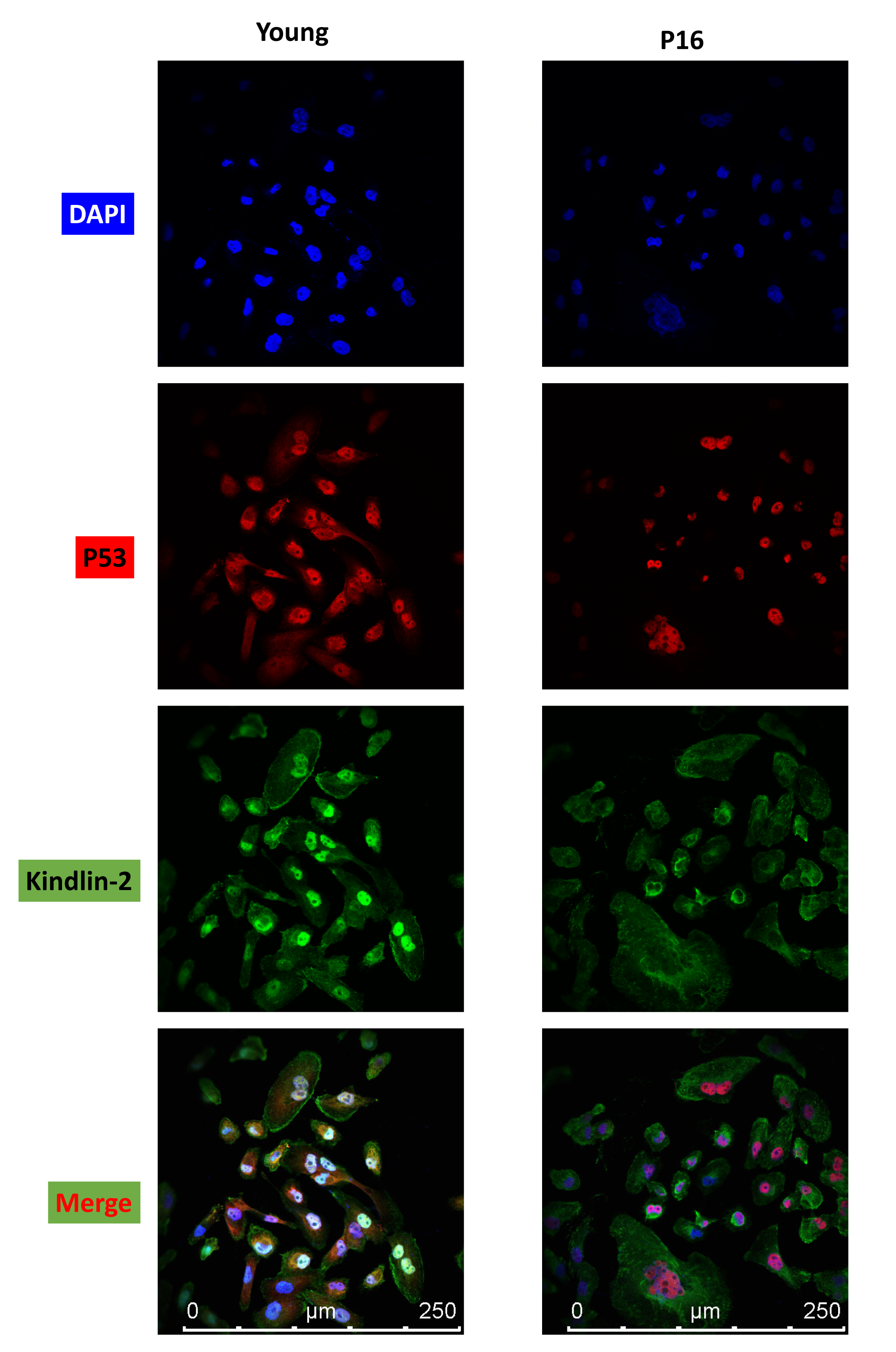

Supplement: Supplementary file 2 — Supplementary Figure 2 [file 41419_2019_1774_MOESM2_ESM.tif]

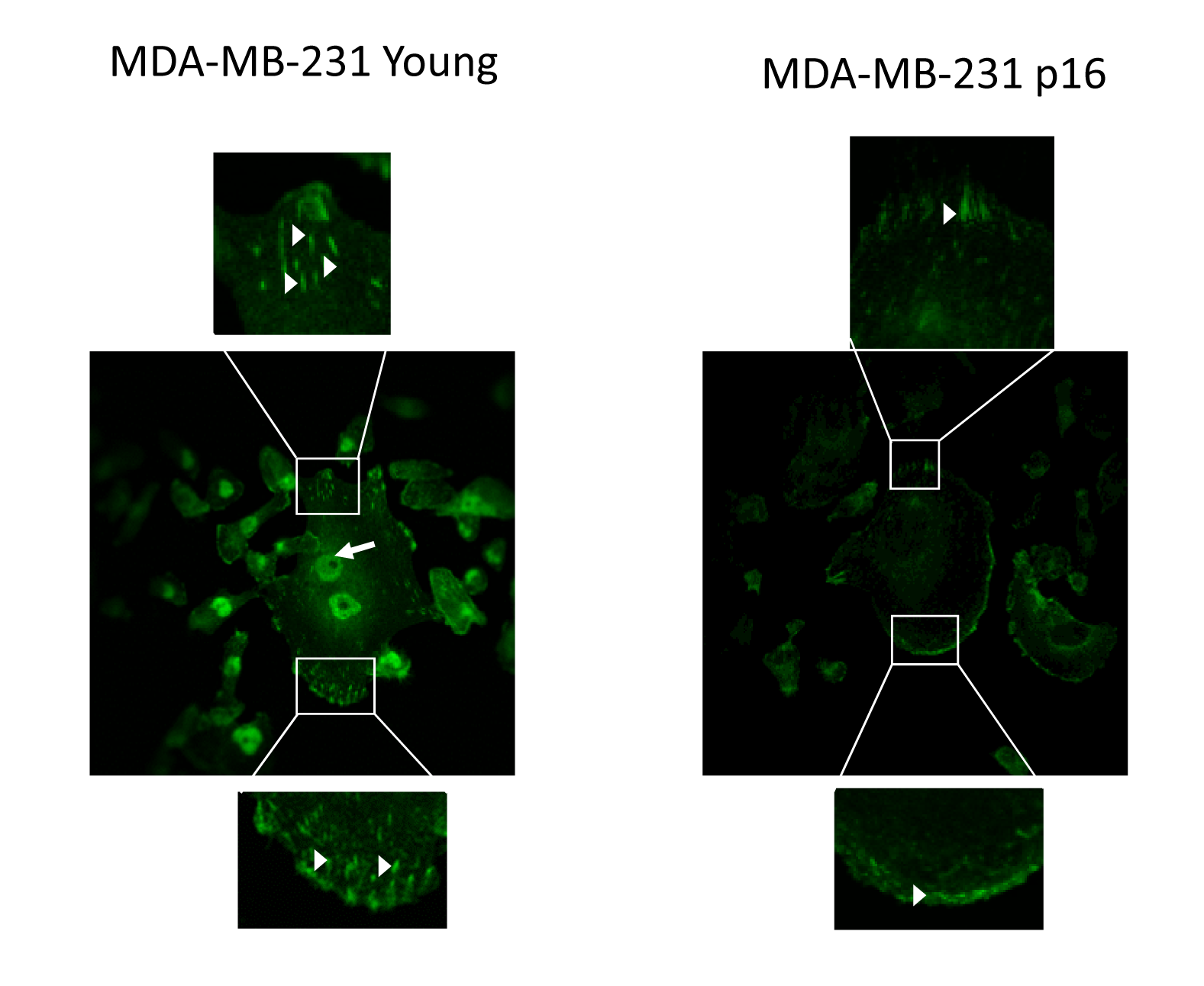

Supplement: Supplementary file 3 — Supplementary Figure 3 [file 41419_2019_1774_MOESM3_ESM.tif]
