# Supplementary material for: The Kindlin2-p53-SerpinB2 signaling axis is required for cellular senescence in breast cancer
Source: Cell Death Dis. 2019 Jul 15;10(8):539. doi: 10.1038/s41419-019-1774-z (PMC6629707; doi:10.1038/s41419-019-1774-z)
Supplement: Supplementary file 4 — Legends for Supplementary Figures [file 41419_2019_1774_MOESM4_ESM.docx]

**Legends for Supplementary Figures**

**Supplementary Figure 1 in support of Figure 3.** (A) Western blots with Kindlin-2 and SerpinB2 antibodies on protein lysates from parental MDA-MB-231 cells (231), control (Scram) and (A) K2-deficient-1 and 2 (K2-KD-1 and K2-KD-2). Anti-β-Actin was used as a loading control. (B) Western blots with SerpinB2 antibody on protein lysates from K2-deficient MDA-231 (231-KO), K2-deficient MDA-231 with SerpinB2-knockdown clone 1 (SerpinB2-KD-1) and K2-deficient MDA-231 with SerpinB2-knockdown clone 2 (SerpinB2-KD-2). Anti-β-Actin was used as a loading control. (C) Western blots with p21 antibody on protein lysates from K2-deficient MDA-231 (231-KO), K2-deficient MDA-231 with p21-knockdown clone 1 (p21-KD-1) and K2-deficient MDA-231 with p21-knockdown clone 2 (p21-KD-2). Anti-β-Actin was used as a loading control.

**Supplementary Figure 2** **in support of Figure 4E.** Representative confocal microscopy images of immunofluorescence staining of early passage (Young, Left panels) or senescent (P16, Right panels) MDA-MB-231 cells that were stained for Kindlin-2 (Green), p53 (Red). Nuclei were counter stained with DAPI (Blue).

**Supplementary Figure 3 in support of Figure 4E.** Representative confocal microscopy images of immunofluorescence staining of early passage (Young) or senescent (P16) MDA-MB-231 cells that were stained for Kindlin-2 (Green). White arrows point to the localization of Kindlin-2 in the nucleus of young but not senescent cells. In the enlarged boxes with higher magnifications the white arrowheads point to the localization of Kindlin-2 to focal adhesions in both young and senescent cells.
